# Iron-loaded activated carbon cloth as CDI electrode material for selective recovery of phosphate

**DOI:** 10.1007/s11356-024-35444-7

**Published:** 2024-11-06

**Authors:** Tanzila Sharker, Jayaruwan G. Gamaethiralalage, Qiyang Qu, Xinxin Xiao, Jouke E. Dykstra, Louis C. P. M. de Smet, Jens Muff

**Affiliations:** 1 Department of Chemistry & Bioscience, Aalborg University, Niels Bohrs Vej 8, 6700 Esbjerg, Denmark; 2 Laboratory of Organic Chemistry, Wageningen University, Stippeneng 4, 6708 WE Wageningen, The Netherlands; 3 Department Department of Chemistry & Bioscience, Aalborg University, Fredrik Bajers Vej 7H, 9220 Aalborg, Denmark; 4 Department of Environmental Technology, Wageningen University, Bornse Weilanden 9, 6708 WG Wageningen, The Netherlands

**Keywords:** Activated carbon cloth, Iron oxide, Phosphate recovery, Chemical desorption, Capacitive deionisation (CDI)

## Abstract

This study investigated the efficacy of oxidised iron-loaded activated carbon cloth (Fe-ACC) for selective recovery of phosphorous. The capacitive deionisation (CDI) technology was employed, for rapid removal of phosphate, with the aim of reducing the reliance on high alkalinity environment for the regeneration of Fe-ACC electrode. Multiple experimental parameters, including applied potential, pH, and co-existing ions, were studied. Additionally, the CDI system was tested on a real water matrix (Lake Ormstrup, Denmark) to elucidate the electrodes’ performance on selective recovery of phosphate. About 69 ± 10% of the adsorbed phosphate were released at pH 12 via pure chemical desorption, which was ~ 50% higher than that at pH 9. The CDI system successfully demonstrated the selective removal of phosphate from the lake water. It reduced the concentration of phosphate from 1.69 to 0.49 mg/L with a 71% removal efficiency, while the removal percentages of other anions, namely chloride, sulphate, bromide, nitrite, nitrate, and fluoride, were 10%, 7%, 1%, 1.5%, 4%, and 7%, respectively.

**Supplementary information:**

The online version contains supplementary material available at 10.1007/s11356-024-35444-7.

## Introduction

Excessive phosphate levels in water bodies from overuse of phosphorus fertilisers can cause eutrophication, collapsing ecosystems (He et al. 2022b, a). Global efforts in mitigating this have led to stricter regulations on phosphate emissions. The EU Water Framework Directive (WFD) limits phosphorous discharge to below 1 to 2 ppm of P, based on the water body’s sensitivity (Wang et al. 2018; Pap et al. 2020; Zhang et al. 2022a). Despite its adverse impacts, phosphorus remains vital in agriculture, highlighting the need for recycling techniques (Gamaethiralalage and de Smet 2023).

Current phosphate removal methods include biological treatment, ion exchange, chemical precipitation, and adsorption. Major challenges in these processes are the lack of selectivity and efficiency over other ions such as chloride, fluoride, bromide, nitrate, nitrite, and sulphate. Biological and chemical treatments generate excessive sludge requiring appropriate disposal (Liu et al. 2018). Physical methods, such as electrodialysis and reverse osmosis, are expensive or ineffective due to poor phosphate selectivity (Bouamra et al. 2018; Li et al. 2021). Adsorption, however, is more efficient, cost-effective, and sludge-free (Velusamy et al. 2021; Zhang et al. 2022a). The effectiveness of adsorption depends on surface charge, electrostatic interactions, chemical bonding, and surface area of the sorbents (Liu et al. 2018; Almasri et al. 2019; Zhang et al. 2022a). Thus, a careful adsorbent selection ensures superior pollutant removal, selectivity, reusability, and stability across various conditions.

Recently, capacitive deionisation (CDI) has emerged as an effective solution for selectively removing charged species. CDI operates on electrostatic interactions and capacitive storage principles. In CDI, a pair of capacitive electrodes, often carbon-based, is placed inside a cell separated by a non-conductive spacer, allowing solution flow. Applying an electric potential or a current across the electrodes attracts ions to the oppositely charged electrodes (Tang et al. 2019; Gamaethiralalage et al. 2021; Wang et al. 2021). Ion recovery through CDI can occur through electric double layer capacitance (EDLC) of porous carbon electrodes, pseudo capacitance of the carbon-redox active composite, or ion intercalation mechanism (Tang et al. 2019). Thus, significant efforts focus on developing high-performance electrode materials. Carbon materials such as activated carbon (AC) powders, AC cloth/fibres, carbon nanotubes, graphene, or graphene oxide possess several key competences for application in CDI due to their high specific surface area, porous structure, good electrical conductivity, chemical stability, hydrophilicity, and ease of preparation and assembly (Thamilselvan et al. 2016; Wu et al. 2019).

Activated carbon cloth (ACC) is a promising material with a substantial surface area of up to 2500 m^2^/g, providing numerous sites for ion adsorption and storage (Geng et al. 2023). Its hierarchical pore structure enhances ion transport and its high-water wettability ensures effective ion adsorption (Geng et al. 2023; Zhang et al. 2023). ACC’s high electrical conductivity of up to 100 S/cm reduces ohmic resistance, lowering the CDI system’s energy consumption (Ye et al. 2016; Geng et al. 2023). Moreover, ACC is resistant to corrosion and oxidation, thus ensuring the durability of the material (Geng et al. 2023).

ACC can be modified or functionalised to selectively target ions such as phosphate (Tang et al. 2019; Liu et al. 2020; Zhang et al. 2022a). Extensive research (Mahardika et al. 2018; Hilbrandt et al. 2019; Zhang et al. 2022a, b; Dai et al. 2023; Li et al. 2023; Zhao et al. 2023) in environmental technology highlights iron oxides/hydroxides as key phosphate adsorbents due to their non-toxicity, cost-effectiveness, and high phosphate affinity. Phosphate binds to iron oxide adsorbents via Fe–P inner-sphere complexes and ligand exchange processes (Bäumler et al. 2021; Spicher et al. 2023). However, regenerating the active binding sites remains challenging. Currently, chemical desorption with sodium hydroxide (NaOH) at high pH (≥ 10) is found to be the most effective method for phosphate desorption from iron oxide (Ajmal et al. 2018; Hou et al. 2020; He et al. 2023).

The primary goal of this work is exploring the feasibility of using an electrical field for regenerating the iron-oxide–carbon-cloth composite materials used for phosphate binding, thus reducing or eliminating the need for high alkaline environments, an approach that has not been reported previously to the best of our knowledge. Within this context, iron-loaded activated carbon cloth (Fe-ACC) was first tested for phosphate adsorption using both passive adsorption as well as electro-assisted adsorption via CDI, while varying experiment parameters such as applied potential, pH, and co-existing ions. Additionally, the electrode material was evaluated using real lake water samples from Lake Ormstrup, Denmark. The findings of this work aim to provide insights into phosphate removal mechanisms and optimize CDI processes for water treatment.

## Materials and methods

### Materials

Sodium phosphate monobasic (NaH_2_PO_4_, ≥ 99%), sodium hexacyanoferrate decahydrate (Na_4_[Fe(CN)_6_∙10H_2_0], ≥ 99%), polytetrafluoroethylene (PTFE, 60% weight in H_2_O), nitric acid (HNO_3_, 70%), and sodium hydroxide (NaOH, ≥ 99%) were purchased from Sigma Aldrich. Nickel chloride hexahydrate (NiCl_2_∙6H_2_O, > 98%) was procured from Acros Organics. Sodium chloride (NaCl, > 99%) was obtained from VWR. Sulphuric acid (H_2_SO_4_, 95–98%) was bought from Honeywell Fluka™. Iron (II) sulphate heptahydrate (FeSO_4_∙7H_2_O, 99 + %) was obtained from Aldrich. The activated carbon cloth (ACC) was purchased from Kynol, Germany. The chemicals were reagent grade and used as received without any further purification. Milli-Q water (18.2 MΩ∙cm, Milli-Q Integral 3 system, Millipore, France) was used to prepare all solutions. Origaflex OGF05A (Origalys, France) potentiostat was used to run all experiments. A Metrohm 913 pH Meter (Metrohm, Switzerland) was used to measure the pH. A Masterflex hose (96400–15, Cole-Parmer, USA) and a Maserflex L/S peristaltic pump (07557–02, Cole-Parmer, USA) were used as a part of the CDI system.

### Analytical techniques—IC and ICP

The concentration of different ions/elements in the solution was assessed both prior to and following the adsorption and desorption procedures via Fe-ACC electrode, employing ion chromatography (Metrohm BV, The Netherlands) and inductively coupled plasma (PerkinElmer Avio 500 ICP-OES). The IC was operated with a carbonate/bicarbonate eluent system and a sulfuric acid regeneration solution, using a Metrosep A Supp 19–100/4.0 column.

### Preparation of electrodes

ACC was modified using a similar method developed by Chen et al. (2007). Two pieces of ACC (10 cm × 10 cm each), weighing a total of 7 g, were immersed in a 250 mL acid mixture consisting of concentrated ${\text{HNO}}_{3}$ and ${\text{H}}_{2}{\text{SO}}_{4}$ (1:1) for 2 h under constant magnetic stirring at room temperature. Afterwards, the acid-treated ACC was thoroughly washed with deionised water until the rinsing water reached a neutral pH, then dried in an oven at 120 °C. The oxidised ACC was then immersed in a 500 mL FeSO_4_∙7H_2_O solution, with an equal mass ratio of ACC and Fe, for 1 h under constant magnetic stirring. Subsequently, 0.2 M NaOH was added dropwise into the solution to achieve a pH of ~ 7 to facilitate iron oxide precipitation. Then, iron oxide-precipitated ACC material (Fe-ACC) was thoroughly washed to remove any residual iron nanoparticles from the surface of ACC and dried in a vacuum oven at 80 °C temperature. A detailed description of synthesis and fabrication of the nickel hexacyanoferrate (NiHCF) electrode can be found elsewhere (Singh et al. 2020).

### Characterisation of Fe-ACC electrode

The scanning electron microscope (SEM – JEOL JAMP-9500F Field Emission Auger Microprobe, and JEOL JSM-5600LV) was used to obtain electron micrographs. X-ray photoelectron spectroscopy (XPS – JEOL JPS-9200 photoelectron spectrometer, JEOL Japan) was used for providing information on the surface morphology and elemental composition of Fe-ACC electrode. The XPS spectra were obtained under ultrahigh vacuum, using a monochromatic Al Kα source at 12 kV and 20 mA. All spectra were corrected with Shirley background fitting and were processed by CASA XPS software (version 2.3.16). The surface area was measured using the Brunauer–Emmett–Teller (BET, NOVAtouch, 2015–2020, Quantachrome instruments, USA). The heating profiles for the BET measurements were set at 250 °C for pristine ACC and 110 °C for Fe-ACC. Streaming potential analysis was performed using an electrokinetic analyser SurPASS (Anton Paar GmbH, Graz, Austria) to study the effect of pH on the electrical charge of the electrode. An automatic titration of the 1 mM KCl reference solution with 0.1 M HCl and 0.1 M NaOH was used for the determination of point of zero charge (PZC). Raman spectroscopy was performed for determining the functional groups of the modified Fe-ACC electrode. A Raman spectrometer MarqMetrix All-In-One (AIO) (Seattle, WA 98103, USA), equipped with a laser with an excitation wavelength of 785 nm and manually adjustable power (from 100 to 450 mW), was employed for the acquisitions.

### Electrochemical impedance spectroscopic (EIS) analysis of Fe-ACC electrode

To explore the electrochemical properties such as specific capacitance and resistance of the electrodes, cyclic voltammetry (CV) and electrochemical impedance spectroscopic (EIS) analysis were conducted using a three-electrodes system. The setup was consisting of a working electrode (ACC or Fe-ACC), Pt mesh counter electrode, and potassium chloride (KCl (aq)) saturated Ag/AgCl reference electrode. The study was performed in a 1 M ${\text{NaH}}_{2}{\text{PO}}_{4}$ electrolyte solution having a pH of 5. The EIS measurement was taken in the frequency range of 10^5^ to 0.1 Hz, at an open circuit potential (OCP) of − 0.072 V.

### Phosphate selective properties of Fe-ACC material

The Fe-ACC electrodes were studied for their selective adsorption capabilities of phosphate and various interfering ions through passive adsorption experiments. Pristine ACC was used for control experiments. Synthetic stock solutions were prepared using various combinations of equal amounts of monovalent phosphate and interfering anions (${\text{H}}_{2}{\text{PO}}_{4}^{-}$/${\text{Cl}}^{-}$, ${\text{H}}_{2}{\text{PO}}_{4}^{-}$/${\text{SO}}_{4}^{2-}$, ${\text{H}}_{2}{\text{PO}}_{4}^{-}$/${\text{NO}}_{3}^{-}$, and ${\text{H}}_{2}{\text{PO}}_{4}^{-}$/${\text{Cl}}^{-}$/${\text{SO}}_{4}^{2-}$/${\text{NO}}_{3}^{-}$) at concentrations ranging from 0 to 500 ppm. Approximately 50 mg of ACC was introduced into Falcon™ tubes, each containing 50 mL of the prepared stock. These Falcon™ tubes, containing the ACC material and stock solutions, underwent agitation in a shaker (Innova 4080 incubator shaker, New Brunswick Scientific) operating at 100 rpm and room temperature for a duration of 24 h.

Subsequently, the solution in each Falcon™ tube was carefully collected after a gravity filtration process to eliminate any residues from the carbon cloth. The concentrations of phosphate and other interfering anions in the solution were determined both before and after the adsorption process, utilising IC. All experiments were conducted in triplicate and in randomised order.

The amount of adsorbed ions by the Fe-ACC electrode (Q [mg/g]) was determined using Eq. 1 (Ajmal et al. 2018):

1 $$Q= \frac{\left({C}_{i }- {C}_{f}\right) \bullet V}{m}$$

where *C*_*i*_ (mg/L) and *C*_*f*_ (mg/L) are the initial and final concentrations of the ion in the electrolyte, respectively; *V* (L) and *m* (g) are the volume of electrolyte and mass of the electrode, respectively.

Additionally, the ion selectivity ($\rho$) of the Fe-ACC material was calculated using Eq. 2 (Gamaethiralalage et al. 2021):

2 $$\rho = \frac{\begin{array}{c}\frac{{C}_{i,in} -{ C}_{i,f}}{{C}_{i,in}}\\ \end{array}}{\begin{array}{c}\frac{{C}_{j,in} - {C}_{j,f}}{{C}_{j,in}}\\ \end{array}}$$

where *C*_*i*,*in*_ (mg/L) and *C*_*i*,*f*_ (mg/L) correspond to the initial and final concentrations of target ion (e.g., phosphate), respectively; *C*_*j*,*in*_ (mg/L) and *C*_*j*,*f*_ (mg/L) represent the initial and final concentrations of the competing ion, respectively.

### Fe-ACC regeneration via chemical desorption

To study the regeneration process of Fe-ACC material, at first, phosphate ions were adsorbed to the Fe-ACC via passive adsorption for 24 h. A 500 ppm synthetic solution containing equal concentration of monovalent phosphate and chloride ions was used for the passive adsorption process. The passive adsorption was conducted at native pH of the solution, which was 5. Subsequently, NaOH was used as the desorption agent, and varying alkaline conditions, with pH levels ranging from 9 to 12, were investigated for the regeneration of Fe-ACC sorbent. The duration of desorption experiments was 4 h, under mild magnetic stirring. All experiments were performed in triplicate to ensure the reproducibility of the results, and the samples were analysed using ICP.

### Phosphate removal via CDI process

**Fig. 1:**
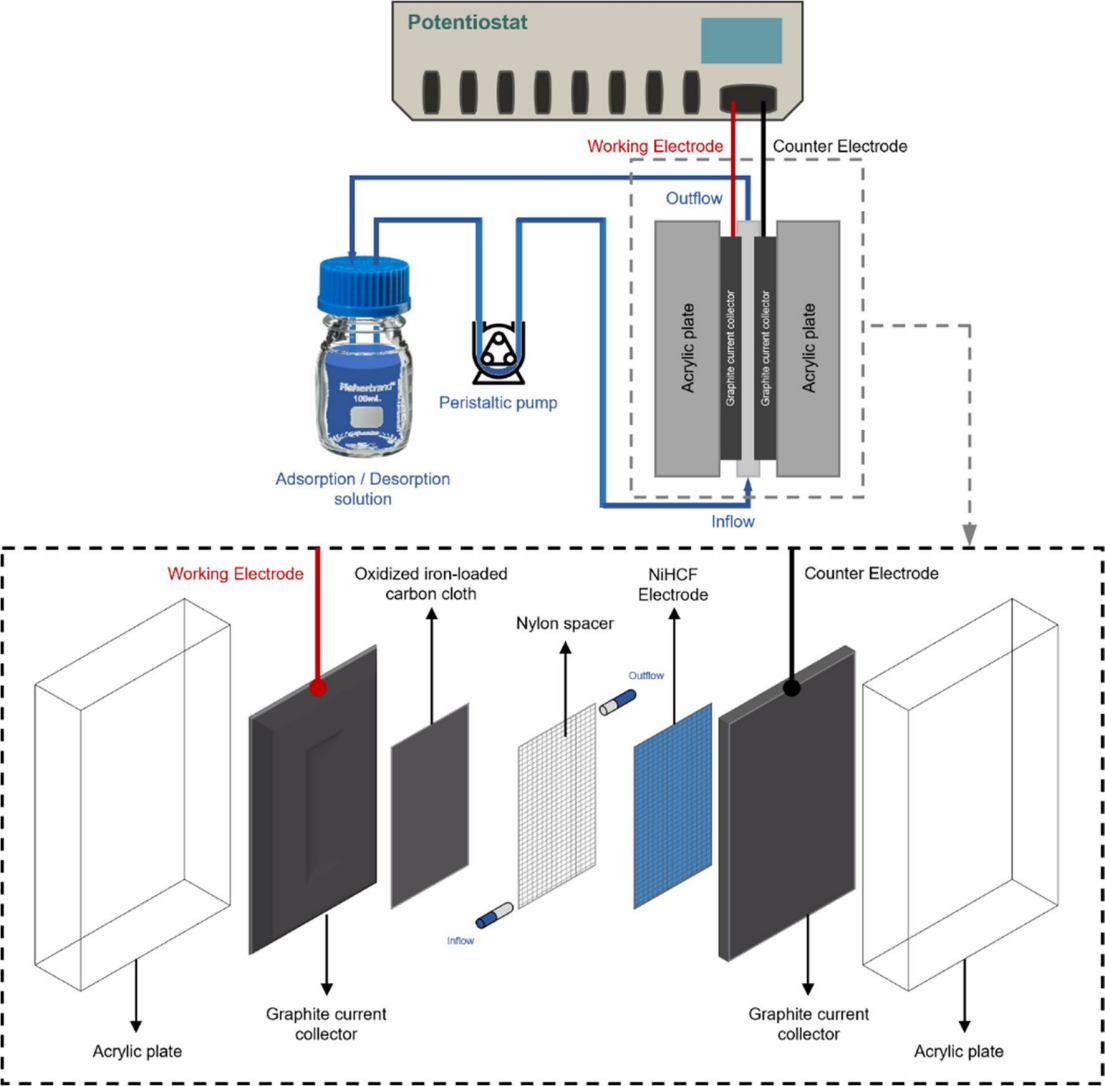
Schematic of the CDI cell consisting of acrylic cases, graphite plates as current collectors, a Fe-ACC working electrode, nylon mesh spacer and turbulence promoter, and a NiHCF counter electrode

To remove the phosphate using CDI, a two-electrode configuration was utilised. The CDI system consisted of a Fe-ACC working electrode and NiHCF counter electrode, each placed on a graphite current collector and separated by a nylon spacer channel. The complete cell assembly is depicted in Fig. 1. The Origaflex OGF05A potentiostat was used for the CDI system.

The electro-assisted adsorption of phosphate was conducted at + 0.3 V applied potential, for 1 h, in a 500 ppm electrolyte solution containing equal concentration of monovalent phosphate and chloride ions. Before continuing with the desorption experiment, CDI cell was rinsed by flushing deionised water (DI) through the cell for 5 min. Subsequently, electro-chemical desorption was conducted, for 1 h, in a NaOH electrolyte of pH 9, at various applied potentials, including open circuit potential (OCP), –1.0 V and –3.0 V. The electrode’s performance in terms of phosphate adsorption capacity, selectivity, and regeneration ability using the CDI system was evaluated over five cycles. The rinsing step involving DI water was also incorporated before switching to the next cycle. All experiments were performed in duplicate and randomised order. The samples were analysed using ion chromatography.

### Phosphate removal from lake water using CDI system

Apart from synthetic salt solutions, lake water (Ormstrup Lake, Denmark) was also used for demonstrating the efficacy of phosphate removal process via Fe-ACC electrode in a classic CDI cell system. In terms of selective phosphate removal, it is essential to consider other competitive species present in the water. Table 1 represents the Ormstrup Lake water anions composition and the ratio of the anions with respect to phosphate ion, which was determined through IC analysis. The pH of the lake water was 6.7. The electro-assisted adsorption in two-electrode CDI system was conducted at + 0.3 V cell potential for 1 h. Subsequently, the electro-chemical desorption was performed at –1.0 V applied potential for 1 h, in a NaOH electrolyte of pH 9. The adsorption and desorption experiments were conducted in duplicate and tested up to five cycles. The samples were analysed using IC for determining the anion concentration prior and post-treatment.

**Table 1:** Anion composition of Ormstrup Lake (Denmark) water

Anions (pH 6.7)	Concentration [mg∙L^−1^, (mM)]	Molar concentration ratio [anions / H_2_PO_4_^−^]
${\text{H}}_{2}{\text{PO}}_{4}^{-}$	1.69 (0.017)	1
${\text{Cl}}^{-}$	27.09 (0.76)	45
${\text{Br}}^{-}$	0.19 (0.0023)	0.14
${\text{NO}}_{2}^{-}$	0.23 (0.0051)	0.30
${\text{NO}}_{3}^{-}$	0.33 (0.0054)	0.32
${\text{F}}^{-}$	0.11 (0.0056)	0.33
${\text{SO}}_{4}^{2-}$	3.40 (0.035)	2

## Results and discussion

### Characterisation of Fe-ACC

**Fig. 2:**
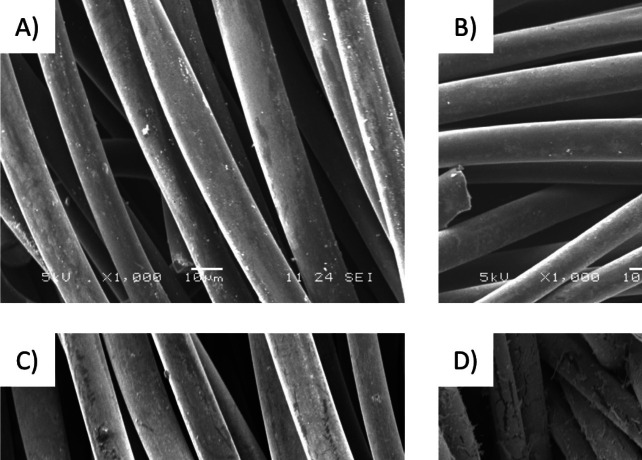
SEM images of **A** pristine, **B** iron-loaded, **C** oxidised, and **D** oxidised and iron-loaded carbon cloth

Figure 2 shows electron micrographs (SEM) of the different stages of Fe-ACC preparation. In a nutshell, Fig. 2A depicts the pristine carbon cloth, 2B iron-loaded carbon cloth without any pre-oxidation, 2C oxidised carbon cloth prior to the iron-loading step, and 2D oxidised and iron-loaded carbon cloth (Fe-ACC). Evidently, the pre-oxidation process enhances the iron-loading process, whereas directly subjecting the carbon cloth to the iron-loading process yields minimal results, which can be seen in Fig. 2B vs. 2D. The initial oxidation process generates surface and pore-bound, oxygen-containing functional groups on the carbon cloth, significantly enhancing the efficiency of the subsequent iron-loading step. Moreover, the XPS analysis provided the surface chemistry of the electrode up to 5–10 nm. The results showed that the surface of pristine ACC consists of 5% oxygen (O) and 95% carbon (C), whereas the iron-loaded into pre-oxidised ACC consists of 59% oxygen (O), 29% iron (Fe), and 12% carbon. These results support the successful integration of iron oxide particles into the surface and pores of oxidised ACC. These immobilised iron particles can act as anchoring sites for specific interactions, which could occur on any accessible surface. Therefore, the subsequent experiments were carried out using the Fe-ACC material.

For pristine ACC, the Raman spectroscopic plot (Figure S1) shows a D-band at ~ 1300 cm^−1^ associated with defects and disorder in the carbon structure and G-band at ~ 1600 cm^−1^ related to graphitic or sp2-bonded carbon atoms. In the case of Fe-ACC (Figure S1), in addition to the peaks at ~ 1325 cm^−1^ and ~ 1600 cm^−1^ corresponding to the graphitic nature and disorder in the ACC, an additional peak is observed at ~ 625 cm^−1^, associated with the iron oxide particles. Additionally, the pristine ACC and Fe-ACC possess a surface area of 1330 m^2^/g and 1177 m^2^/g, respectively. The observed decrease in surface area could be the result of harsh chemical oxidation treatment of ACC.

Furthermore, the PZC of the electrode was determined to be at approximately pH 11 (Figure S2). Below this pH, the electrode surface tends to become positively charged, attracting negatively charged ions such as phosphate, which enhances phosphate removal. Conversely, above the PZC, the surface becomes negatively charged, facilitating the regeneration of the electrode material by releasing the adsorbed ions.

### EIS and CV study of Fe-ACC

The ACC went through a chemical pre-treatment and subsequent iron impregnation process for enhancing the adsorption capacity and selectivity towards phosphate. Hence, it is important to assess the electrical behaviour of the modified electrode for application purposes. The specific capacitance of pristine ACC and Fe-ACC were calculated to be 661 µF/cm^2^ and 295 µF/cm^2^, respectively, based on CV curves obtained at a scan rate of 5 mV/s (Figure S3). This indicates that the specific capacitance was reduced by approximately twofold as a result of the modification of ACC.

**Fig. 3:**
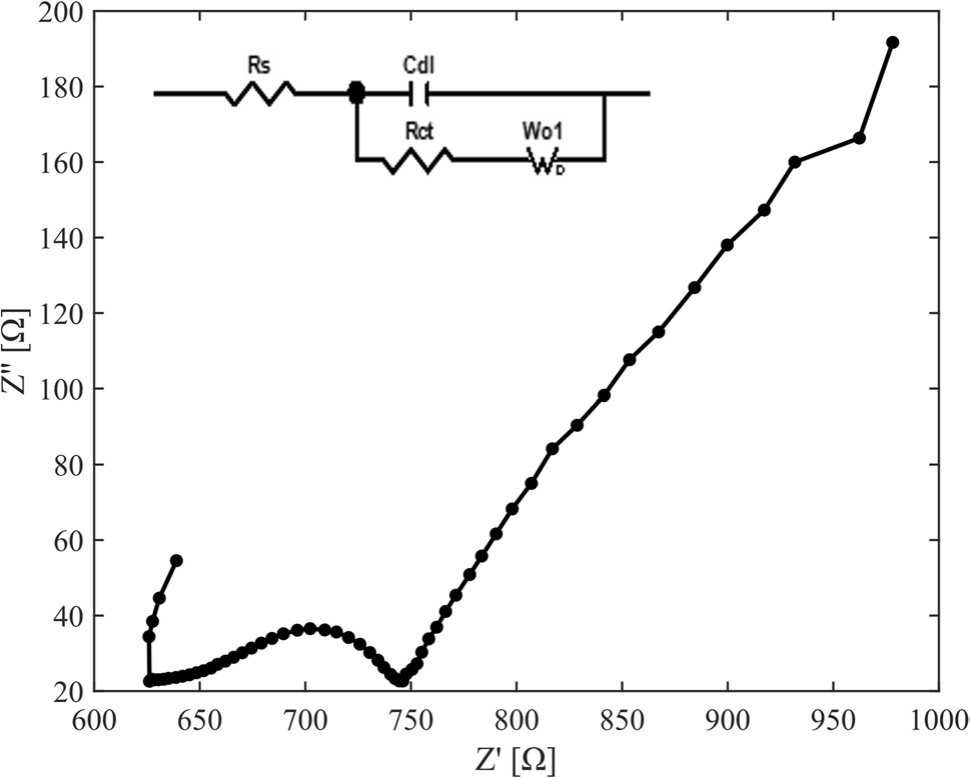
Nyquist plot of Fe-ACC and fitted equivalent circuit model

Additionally, the EIS study was performed to characterise Fe-ACC electrode for CDI applications. Nyquist plots shown in Fig. 3 were used to analyse the EIS data by fitting it to an equivalent circuit model. Nyquist plots consist of the real part of the impedance (Z´) versus the negative imaginary part of the impedance (Z´´) as a function of frequency. In this context, Z´ and Z´´ represent the resistance and the capacitance of the electrode and electrolyte interface, respectively. To extract information about capacitance from EIS data of Fe-ACC, the fitted model shown in Fig. 3 was used. The circuit consists of a solution resistance (Rs), a double layer capacitance (Cdl), charge transfer resistance (Rct), and Warburg impedance (Wo). From the fitted model, the Rs and Rct were extracted to be 639 Ω and 104 Ω, respectively. The value of Rct corresponding to the semi-circle in the Nyquist plot confirms the existing resistance to charge accumulation at the electrode interface, which resulted from the electrochemical reaction or adsorption process. Note that Rct is inversely proportional to specific capacitance, meaning a higher specific capacitance generally results in a lower Rct. The Warburg impedance, which accounts for diffusion or mass transport of ions in the solution or pores, is negligible in this case due to the high concentration of the electrolyte (1 M sodium phosphate monobasic electrolyte solution), which minimises diffusion limitations.

Generally, the capacitance is linearly proportional to the electrode’s active surface area. The texture of the electrodes strongly influences the surface charge density and ion distribution in the electrical double layer (EDL) (Dujearic-Stephane et al. 2021). Pristine ACC (Kynol, Germany) has a surface area of 1330 m^2^/g, obtained from BET analysis. On the other hand, pre-treatment of ACC with strong oxidising agents such as concentrated sulfuric acid and nitric acid results in the formation of carboxylic acid functional groups on the surface (Azhagapillai et al. 2021; Dubey et al. 2021). These functional groups can enhance the hydrophilicity and surface charge density of the ACC. However, different studies (Wepasnick et al. 2010; Azhagapillai et al. 2021) suggested that pre-treatment with strong oxidising agents can decrease the specific capacitance due to the loss of active sites and the corrosion of carbon structures. The results obtained from BET analysis and electrochemical measurements of ACC and Fe-ACC are consistent with those studies, showing a decrease in surface area and specific capacitance following treatment.

### Phosphate removal performance of Fe-ACC

#### Phosphate-selective properties of Fe-ACC

**Fig. 4:**
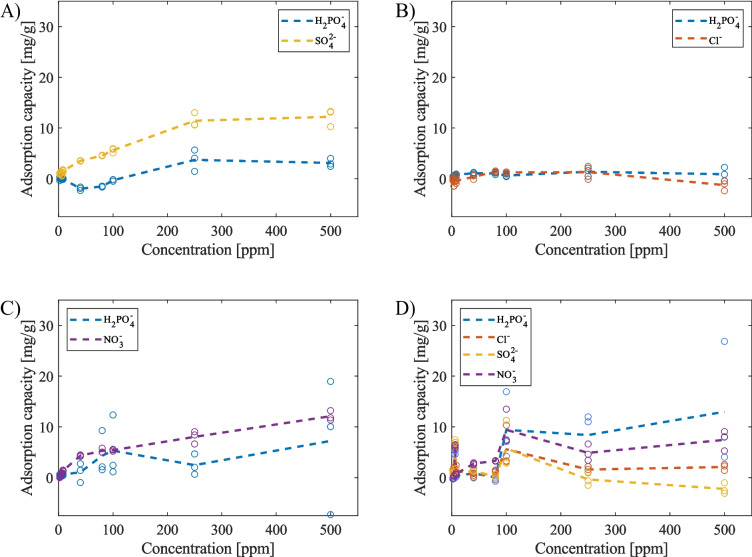
Passive adsorption results from pristine ACC experiments in **A** phosphate (${H}_{2}P{O}_{4}^{-}$) vs. sulphate ($S{O}_{4}^{2-}$), **B** phosphate vs. chloride ($C{l}^{-}$), **C** phosphate vs. nitrate ($N{O}_{3}^{-}$), and **D** phosphate vs. chloride, sulphate, and nitrate

Figure 4 illustrates the phosphate adsorption performance of the pristine carbon cloth across various concentrations (ranging from 0 to 500 ppm) while contending with equal amounts of other competing anions, namely chloride, sulphate, and nitrate. The overarching trend, as observed in all the plots within Fig. 4, consistently portrays a phosphate adsorption capacity that is either lower or on par with the adsorption of the competing anions, indicating that the pristine carbon cloth exhibits a suboptimal phosphate adsorption capacity and lacks selectivity. This outcome can be attributed to the non-specific adsorption mechanism, primarily driven by interactions such as electrostatic forces, van der Waals forces, hydrogen bonding, and hydrophobic interactions (Yang et al. 2018; Saifuddin et al. 2019). Consequently, anions with smaller hydration energies (ΔG_hyd_), like chloride (−340 kJ/mol) and nitrate (−300 kJ/mol), as well as those possessing a higher net charge, such as sulphate (−1080 kJ/mol), proved to be more competitive in occupying the surface and pores of the pristine carbon cloth under these conditions compared to phosphate (−2765 kJ/mol) (hydration energies are from Marcus 1994).

**Fig. 5:**
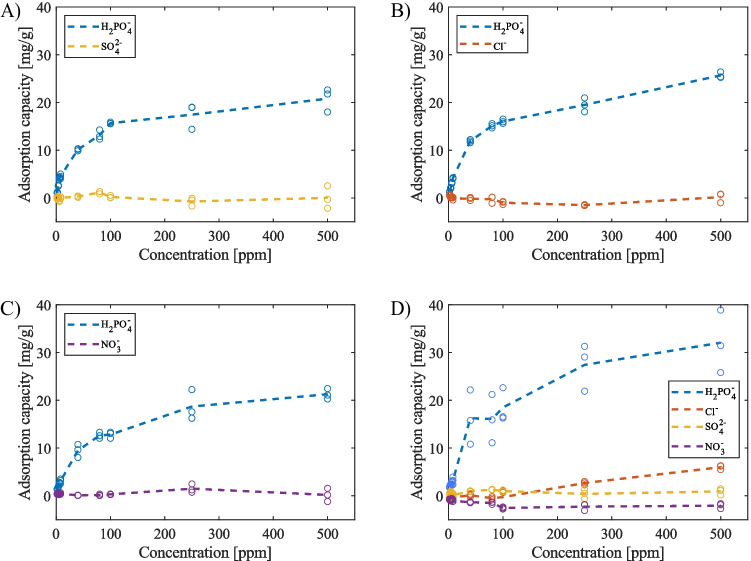
Passive adsorption results from Fe-ACC experiments in **A** phosphate (${H}_{2}P{O}_{4}^{-}$) vs. sulphate ($S{O}_{4}^{2-}$), **B** phosphate vs. chloride ($C{l}^{-}$), **C** phosphate vs. nitrate ($N{O}_{3}^{-}$), and **D** phosphate vs. chloride, sulphate, and nitrate

In contrast, similar trends were consistently observed across all scenarios involving the Fe-ACC, as depicted in Fig. 5A–D. Notably, the phosphate adsorption capacity exhibited a significant advantage over other competing anions across the entire concentration range, spanning from 0 to 500 ppm. The phosphate adsorption capacity showed a substantial increase, starting from 1.2–1.8 mg/g at 2 ppm to a range of 10–12 mg/g at 40 ppm. This suggests the presence of a considerable number of available phosphate adsorption sites within this concentration range. As the concentrations were further raised from 40 to 500 ppm, the curves flattened, indicating a gradual increase in phosphate adsorption capacity that ultimately settled at approximately 20–25 mg/g at 500 ppm. This levelling off indicates that the phosphate adsorption sites on the Fe-ACC were becoming saturated, having reached their maximum capacity.

In addition, the adsorption capacities of the competing anions remained consistently around zero, signifying that the selectivity of the Fe-ACC was exclusively directed towards phosphate. The presence of the competing anions had a negligible effect on the phosphate adsorption capacity of the Fe-ACC, further emphasising its remarkable selectivity.

This phosphate adsorption capacity and selectivity exhibited by the Fe-ACC can be attributed to its strong affinity for phosphate, which is facilitated by the presence of fixed iron oxides/hydroxides on the modified carbon cloth’s surface. This strong affinity between iron and phosphate can be explained utilising the Lewis acid/base theory and the Hard/Soft Acids/Bases (HSAB) principle (Pearson 1968).

Phosphate is classified as a hard base, and typical hard acids like Fe^2+^/Fe^3+^, La^3+^, and Al^3+^ based materials are frequently chosen for the selective removal of phosphate in wastewater treatment (Arai and Sparks 2001; Tanada et al. 2003; Kawasaki et al. 2010; Mahardika et al. 2018; Razanajatovo et al. 2021). Similar to phosphate, anions such as sulphate, carbonate, nitrate, chloride, and fluoride also fall under the category of hard bases. However, their capacity to donate electron pairs (Lewis basicity) to a Lewis acid varies, with phosphate having the highest Lewis basicity, followed by carbonate, sulphate, fluoride, chloride, and nitrate (Wu et al. 2020). This indicates that the coordination between iron and phosphate is more favourable than with other anions. Consequently, the iron tends to coordinate with phosphate even in the presence of anions such as sulphate, chloride, and nitrate in the same aqueous system.

Furthermore, previous studies suggest that the primary mechanism for phosphate adsorption by metal oxides/hydroxides involves inner-sphere complexation through ligand exchange, which entails the replacement of hydroxyl groups on the surface of the metal oxides/hydroxides with phosphate ions, resulting in the formation of coordinate bonds (Antelo et al. 2010; Wang et al. 2014; Li et al. 2016; Wu et al. 2020; Razanajatovo et al. 2021). Notably, many previous studies have observed that metal oxides/hydroxides exhibit increased phosphate adsorption capacity at acidic pH levels compared to basic pH (Liu et al. 2011; Xie et al. 2014; He et al. 2017; Yuan et al. 2018). This indicates that under acidic conditions, the protonated hydroxyl groups are more amenable to being replaced by phosphate ions than under basic conditions. Additionally, the presence of protonated hydroxyl groups and a positively charged surface on the sorbent can attract the negatively charged phosphate ions, facilitating the ligand exchange process (Razanajatovo et al. 2021).

In addition, it is important to note that one phosphate ion can bind to one or two iron atoms through one or two oxygen atoms, rendering the inner-sphere complexation more stable (Arai and Sparks 2001; Wang et al. 2014; Wu et al. 2020). In contrast, when phosphate is present at acidic pH, anions such as chloride, sulphate, and nitrate typically form outer-sphere complexes primarily driven by electrostatic interactions between the anions and the positively charged adsorbent surface (Wu et al. 2020). Comparatively, the interactions in inner-sphere complexes are significantly stronger than simple electrostatic interactions. Consequently, phosphate ions exhibit selective adsorption onto the Fe-ACC, driven by these powerful forces.

The strong interactions involved in inner-sphere complexation, while advantageous for selective adsorption, present a distinct challenge. Unlike ions in outer-sphere complexes, the ions adsorbed through inner-sphere complexation tend to require harsh conditions, such as high alkalinity, to be effectively released from the sorbent material. This can make the reusability of the sorbent material more challenging, as the desorption process becomes more demanding under such conditions. Consequently, the subsequent sections of this study have focused on exploring methods for either eliminating or mitigating the need for harsh environments, thereby enhancing the sorbent material’s reusability.

#### Performance assessment of chemical desorption of phosphate

**Fig. 6:**
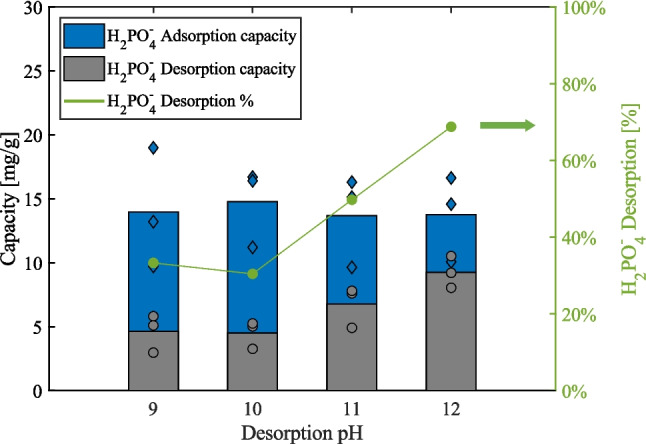
The blue and grey colour bar plots represent the average value of phosphate (${H}_{2}P{O}_{4}^{-}$) adsorption and desorption capacity, respectively, obtained at various pH ranging from 9 to 12. The data from triplicate experiments are shown using blue diamond (adsorption capacity) and grey circle (desorption capacity) marker styles. The green line plot represents the average desorption percentage (%) of the triplicate experiments

The bar plot in Fig. 6 demonstrates the phosphate removal capacity via passive adsorption, chemical desorption data obtained at pH ranging from 9 to 12, and desorption percentage (%). Virtually, 100% selectivity towards monovalent phosphate over chloride ions was obtained during the 24-h passive adsorption. The resulting capacity of the Fe-ACC obtained to be 14 ± 4 mg/g, whereas the maximum desorption achieved was 69 ± 10% at a high alkaline condition (pH 12), whereas a low alkaline environment (pH 9) released 33 ± 6% of the adsorbed phosphate. There was no difference observed between desorption pH 9 and pH 10, whereas pH 11 resulted in 50 ± 4% recovery of adsorbed phosphate.

The concentration of hydroxyl ions at pH 12 is significantly higher compared to pH 9. It plays a role in the phosphate desorption from iron oxide by affecting the surface charge and stability of the complexes. According to studies (Hou et al. 2020; Chen et al. 2023), the point of zero charge (PZC), at which surface charge of iron oxide is zero, ranges from pH 6.8 to pH 9.0, depending on the particle size and surface chemistry of iron oxide. At pH values higher than the PZC, the surface charge of iron oxide is negative, which reduces the electrostatic attraction between phosphate and iron oxide, thus increasing the desorption of phosphate. Furthermore, outer-sphere complexes are weaker and involve water molecules as a bridge between phosphate and iron oxide, thus making them less stable and easily affected by pH changes. On the other hand, the inner-sphere complexes are stronger and involve direct coordination of phosphate ions to the iron molecules through oxygen atoms, hence more stable and less affected by pH changes. A study (Kim et al. 2011) showed that the release of phosphate from the inner-sphere complexes is mainly controlled by the ligand exchange reaction with anions such as carbonate (${\text{CO}}_{3}^{2-}$), bicarbonate (${\text{HCO}}_{3}^{-}$), or silicate among others. The presence of hydroxyl ions can affect the availability and speciation of these anions, through which it can contribute to the ligand exchange reaction. Finally, the released phosphate is more readily soluble in the high alkaline environment.

In conclusion, this investigation, exploring the impact of varying pH on phosphate release and Fe-ACC regeneration, revealed that the desorption was approximately 50% higher at pH 12 than pH 9. However, the 100% desorption of phosphate was unable to obtain due to the existing inner-sphere complexes, which was less sensitive to pH changes.

#### Performance assessment in CDI process

**Fig. 7:**
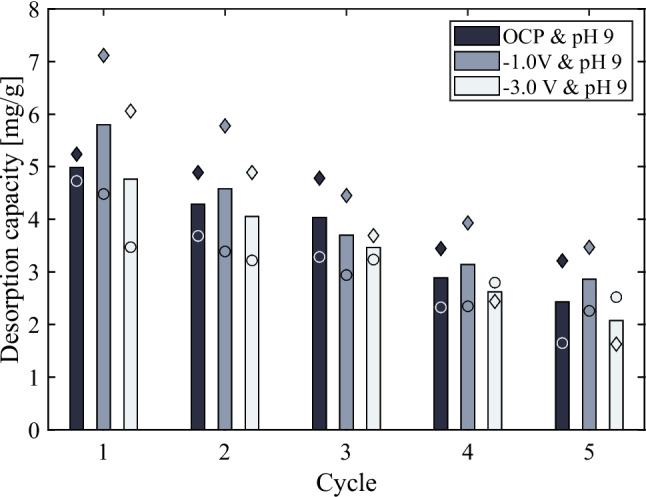
The bar plot represents the average desorption capacity of phosphate at open circuit (OC), − 1.0 V and − 3.0 V cell potentials, in NaOH electrolyte solution of constant pH 9, over five cycles. The diamond and circle marker styles filled with respective colours of the bars stand for the experimental data points over five cycles

To study the overall contribution from the capacitive behaviours of Fe-ACC electrode, the electrosorption and electrochemical desorption of phosphate were conducted. The bar plot shown in Fig. 7 corresponds to the electro-assisted desorption capacity of the phosphate ions. The parameters studied for phosphate recovery and electrode regeneration were open circuit (OC), − 1.0 V and − 3.0 V cell potential, at a constant alkaline NaOH electrolyte of pH 9.

The adsorption data is omitted from Fig. 7 due to the observation that the total amount of ions removed did not align with the electrode’s adsorption capacity. An uncharacteristically elevated adsorption capacity value prompted an examination of the rinsing solution. The analysis revealed that this value encompassed ions adsorbed into the electrode, ions retained in the spacer channel, and on the surface of the carbon cloth. Nevertheless, the revised adsorption capacity of the electrode aligns with the observations made in the “Performance assessment of chemical desorption of phosphate” section. Moreover, the desorption efficiency at pH 9 corresponds consistently with previous observations. Consequently, these CDI experiments demonstrate that at a 0.3 V cell potential, the adsorption of phosphate ions is accelerated, driven by electrical forces, resulting in a faster removal from water. This occurs with only a minor compromise to selectivity when compared to a 24-h passive adsorption process. Figure 7 further suggests that incomplete desorption leads to a gradual decline in electrode capacity. Notably, it indicates that applying a potential during the regeneration phase has a negligible effect, highlighting the ongoing challenge in regenerating Fe-ACC material at low alkaline solutions.

#### Performance assessment in lake water

**Fig. 8:**
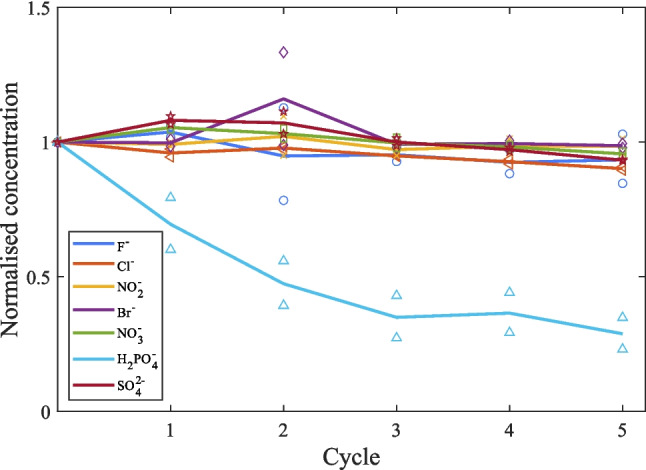
The line plots represent normalised concentration of the average removal of different ions from the lake water (Lake Ormstrup, Denmark), including fluoride (${F}^{-}$), chloride ($C{l}^{-}$), nitrite ($N{O}_{2}^{-}$), bromide (${Br}^{-}$), nitrate ($N{O}_{3}^{-}$), phosphate (${H}_{2}P{O}_{4}^{-}$), and sulphate ($S{O}_{4}^{2-}$) via Fe-ACC electrode in the CDI system, at + 0.3 V cell potential. Different marker styles are used for showing the experimental values corresponding to different ions

Anions in the lake water (Table 1) exist in the following ascending order of concentration: bromide < nitrite < nitrate < fluoride < phosphate < sulphate < chloride. Figure 8 shows the normalised concentration of different anions in the lake water versus the experimental cycles, which follows a steep descending curve for phosphate compared to other anions. During the phosphorus capturing step from the lake water, the CDI system successfully reduced the concentration of phosphate from 1.69 to 0.49 mg/L following the 5th cycle, which was about a 71% removal efficiency. The removal percentages of competitive anions such as chloride, sulphate, bromide, nitrite, nitrate, and fluoride were 10%, 7%, 1%, 1.5%, 4%, and 7%, respectively. This indicates a strong preference for the removal of phosphate ions from lake water. At the 5th cycle, the selectivity of phosphate over chloride, sulphate, bromide, nitrite, nitrate, and fluoride ions were determined to be 5.9, 6.4, 7.3, 7.3, 6.8, and 6.4, respectively. This result successfully demonstrated the efficacy of CDI technology, which utilised a Fe-ACC working electrode and a cell potential of 0.3 V, in achieving selective phosphate removal performance in a real water matrix.

## Conclusion

This study validates that the modification of ACC through chemical oxidation and iron impregnation effectively enhances selectivity towards phosphate ions. The unmodified ACC displayed notably low adsorption capacity and lacked selectivity for phosphate due to nonspecific interactions. Anions with smaller hydration energies (e.g., chloride and nitrate) and those with higher net charges (such as sulphate) were more effective in binding to the pristine electrode surface. In contrast, when considering both passive adsorption and electrosorption processes utilising the modified Fe-ACC electrode, the presence of competitive anions has minimal impact on phosphate selectivity, owing to the fixed iron oxides/hydroxide sites on the electrode.

The enhanced phosphate selectivity in the modified electrode can be attributed to the formation of inner-sphere complexation through ligand exchange processes, aligning with existing literature. While stable inner-sphere complexation interactions are advantageous for selectively adsorbing phosphate, they present a challenge in material regeneration. Consequently, the study explored chemical desorption under different alkaline conditions (pH 9–12) and electrochemical desorption with CDI at various potentials (OCP, − 1.0 V, and − 3.0 V) combined with a low alkaline environment. Chemical desorption at a high alkaline environment (pH 12) resulted in a 50% higher desorption compared to a low alkaline environment (pH 9). Unfortunately, the impact of applied potential on material regeneration remained negligible, further reinforcing the need for high alkaline regenerants. Nevertheless, the Fe-ACC electrode exhibited excellent selectivity and accelerated adsorption at 0.3 V, even when tested in real lake water.

## Supplementary information

Below is the link to the electronic supplementary material.

Supplementary file1 (DOCX 2526 KB)

## Data Availability

The authors declare that the data supporting the findings of this study are available within the paper and its Supplementary Information files. Should any raw data files be needed in another format, they are available from the corresponding author upon reasonable request.
