# Supplementary material for: Iron-loaded activated carbon cloth as CDI electrode material for selective recovery of phosphate
Source: Environ Sci Pollut Res Int. 2024 Nov 6;31(55):63734–46. doi: 10.1007/s11356-024-35444-7 (PMC11602819; doi:10.1007/s11356-024-35444-7)
Supplement: Supplementary file 1 — Supplementary file1 (DOCX 2526 KB) [file 11356_2024_35444_MOESM1_ESM.docx]

**Supporting Information**

**Iron-loaded activated carbon cloth as CDI electrode material for selective recovery of phosphate**

Tanzila Sharker^1,‡^, Jayaruwan G. Gamaethiralalage^1,2,‡^, Qiyang Qu^2^ , Xinxin Xiao^3^, Jouke E. Dykstra^4^, Louis C. P. M. de Smet^2^, Jens Muff ^1,^*

*^1^ Department of Chemistry & Bioscience, Aalborg University, Niels Bohrs Vej 8, 6700 Esbjerg, Denmark*

*^2^Laboratory of Organic Chemistry, Wageningen University, Stippeneng 4, 6708 WE Wageningen, The Netherlands*

*^3^Department Department of Chemistry & Bioscience, Aalborg University, Fredrik Bajers Vej 7H, 9220 Aalborg, Denmark*

*^4^Department of Environmental Technology, Wageningen University, Bornse Weilanden 9, 6708 WG Wageningen, The Netherlands*

** Corresponding authors:* [*jm@bio.aau.dk*](mailto:jm@bio.aau.dk)

^‡^ *These authors contributed equally and share the first authorship*


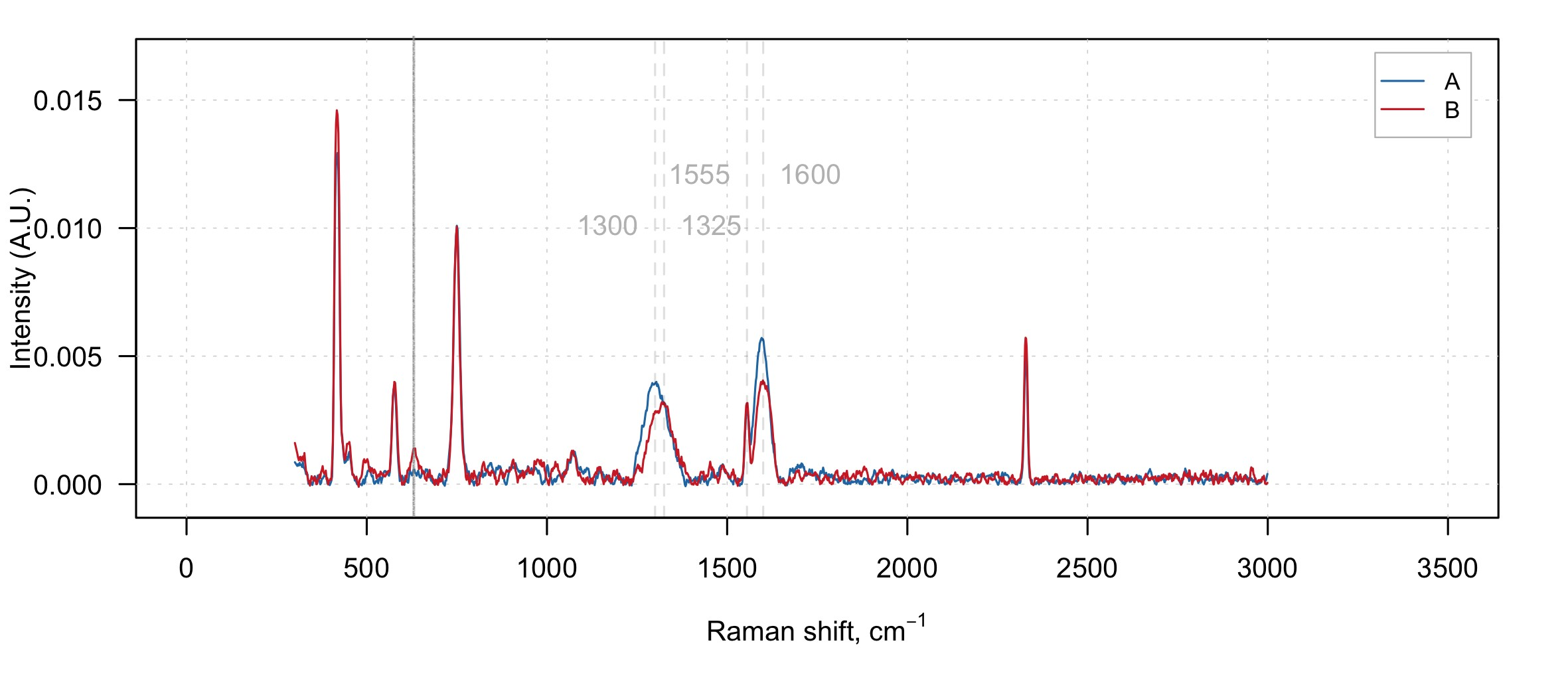


Figure S1: Raman spectroscopy of pristine ACC (A, blue), and Fe-ACC (B, red)

Figure S2: Streaming potential analysis to study the effect of pH dependent surface charge for the determination of point of zero charge of iron oxide loaded activated carbon cloth (Fe-ACC).


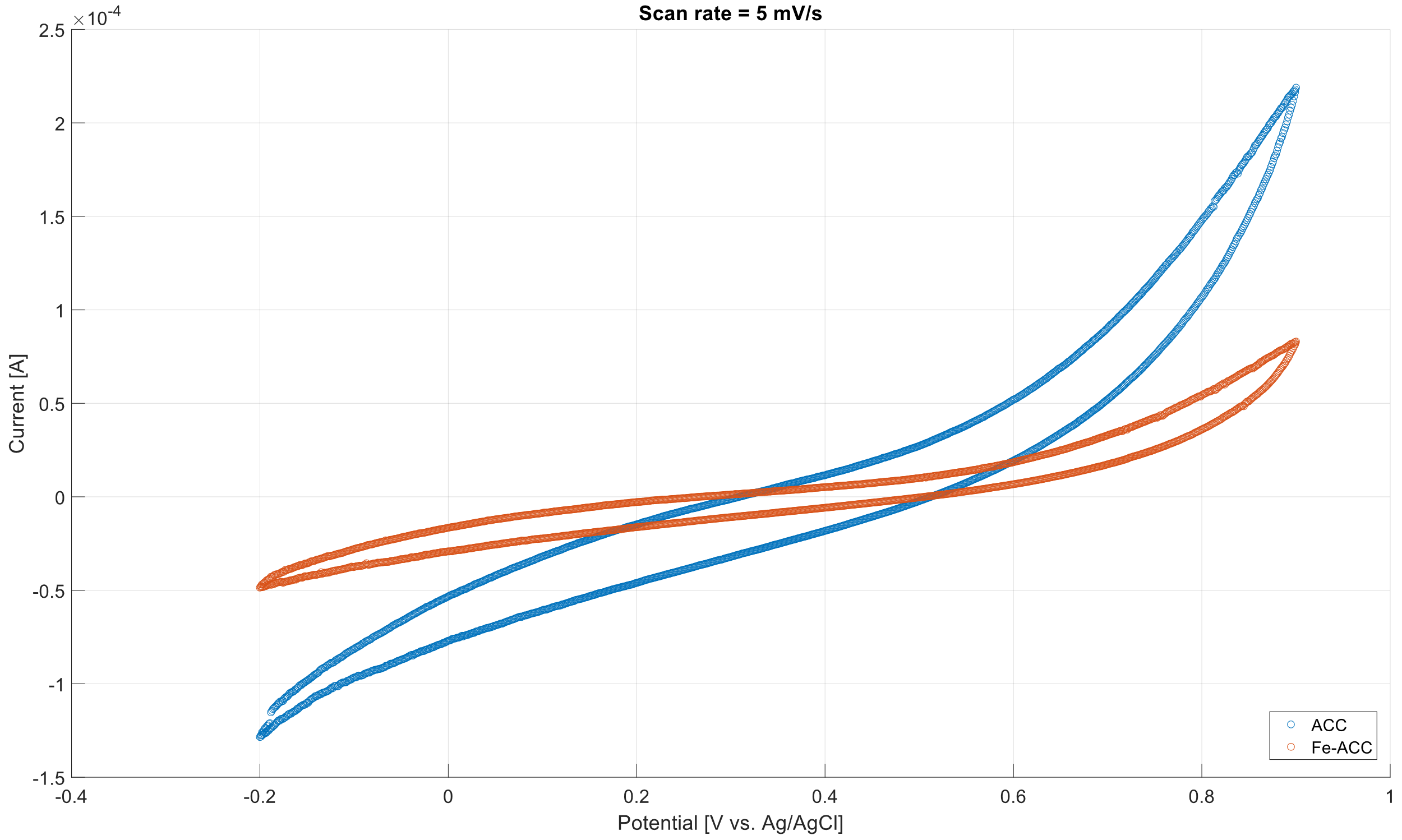


Figure S3: CV curves of pristine-ACC and Fe-ACC obtained at scan rate 5 mV/s, a potential window of -0.2V to 09.V, and in a 1 M NaH_2_PO_4_ electrolyte solution.

The specific capacitances (Cs) of ACC and Fe-ACC were calculated to be 661 µF/cm^2^ and 295 µF/cm^2^, respectively, using the CV curves shown in **Figure S3**. The following equation was used for determining the Cs values.

$$C_{s}=\frac{\int I\left( V \right)dV}{\Delta V*A*\nu}$$

where, $\int I\left( V \right)dV$ is the integral of the current (I) with respect to voltage (V); $\Delta V$ is the potential window (-0.2V to +0.9V); A and $\nu$ are the geometric surface area of the electrode (1 cm^2^) and potential scan rate (5 mV/s), respectively.
